# Erythroid Promoter Confines FGF2 Expression to the Marrow after Hematopoietic Stem Cell Gene Therapy and Leads to Enhanced Endosteal Bone Formation

**DOI:** 10.1371/journal.pone.0037569

**Published:** 2012-05-18

**Authors:** Xianmei Meng, David J. Baylink, Matilda Sheng, Hongjie Wang, Daila S. Gridley, K.-H. William Lau, Xiao-Bing Zhang

**Affiliations:** 1 Division of Regenerative Medicine, Department of Medicine, Loma Linda University, Loma Linda, California, United States of America; 2 Division of Medical Genetics, Department of Medicine, University of Washington, Seattle, Washington, United States of America; 3 Department of Radiation Medicine, Loma Linda University, Loma Linda, California, United States of America; 4 Musculoskeletal Disease Center, Jerry L. Pettis Memorial VA Medical Center, Loma Linda, California, United States of America; Emory University School of Medicine, United States of America

## Abstract

Fibroblast growth factor-2 (FGF2) has been demonstrated to be a promising osteogenic factor for treating osteoporosis. Our earlier study shows that transplantation of mouse Sca-1^+^ hematopoietic stem/progenitor cells that are engineered to express a modified FGF2 leads to considerable endosteal/trabecular bone formation, but it also induces adverse effects like hypocalemia and osteomalacia. Here we report that the use of an erythroid specific promoter, β-globin, leads to a 5-fold decrease in the ratio of serum FGF2 to the FGF2 expression in the marrow cavity when compared to the use of a ubiquitous promoter spleen focus-forming virus (SFFV). The confined FGF2 expression promotes considerable trabeculae bone formation in endosteum and does not yield anemia and osteomalacia. The avoidance of anemia in the mice that received Sca1^+^ cells transduced with FGF2 driven by the β-globin promoter is likely due to attenuation of high-level serum FGF2-mediated stem cell mobilization observed in the SFFV-FGF2 animals. The prevention of osteomalacia is associated with substantially reduced serum Fgf23/hypophosphatemia, and less pronounced secondary hyperparathyroidism. Our improved stem cell gene therapy strategy represents one step closer to FGF2-based clinical therapy for systemic skeletal augmentation.

## Introduction

Osteoporosis, or porous bones, is a disease that leads to an increased risk of fracture. Over 20 million people have osteoporosis in the United States, and approximately 1.3 million people each year suffer a bone fracture as a result of osteoporosis. Current therapies and drugs in development range from bisphosphonates, parathyroid hormone (PTH), RANKL inhibitors or anti-sclerostin antibodies. However, these therapies cause significant side effects after long-term use [1], [2], [3]. Therefore, the development of alternative strategies for efficient treatment of osteoporosis is warranted.

Previous studies have demonstrated that fibroblast growth factor-2 (FGF2; also known as basic FGF or bFGF) is a bone anabolic agent able to promote bone formation by stimulating proliferation and differentiation of mesenchymal stem cells [4], [5], [6]. Systemic administration of recombinant FGF2 protein increases bone formation [7], [8], [9], [10] and promotes fracture repair [11]. FGF2 primarily initiates the growth of new trabeculae and increases trabecular mass and interconnectivity [7], [12]. However, due to a very short in vivo half-life of FGF2, daily injection of large quantities of FGF2 protein is necessary, which is infeasible and expensive in clinical practice. As such, we have taken a hematopoietic stem cell (HSC) gene therapy approach to achieve long-term stable FGF2 expression. After transplantation of mouse bone marrow (BM) Sca-1^+^ hematopoietic stem/progenitor cells that were transduced with a murine leukemia virus (MLV)-based vector expressing a modified *FGF2* gene, we observed substantially enhanced trabecular bone formation at the endosteal surface [13]. The osteogenesis-promoting capacity of FGF2 after transplantation of FGF2-expressing HSCs is superior to other similar osteogenic factors such as bone morphogenic protein 4 (BMP4) [14]. However, the MLV-based HSC gene therapy yields FGF2 levels that are ∼100-fold higher than physiological concentrations in serum, which may increase chances of unintended side-effects, including tumorigenesis. In addition, we also observed an up to a 100-fold variation in FGF2 serum levels in different mice, which is likely due to silencing of the viral long terminal repeat (LTR) and/or clonal effects. Moreover, mice with high-level serum FGF2 developed anemia and osteomalacia [13].

To overcome the limitations of the current therapy and further develop FGF2-based HSC gene therapy into a new strategy for treating severe osteoporosis, we hypothesized that the use of an erythroid promoter to drive the expression of FGF2 might increase local expression at the marrow cavity and decrease the serum FGF2 levels. If this is the case, the confined FGF2 expression to the marrow cavity would promote endosteal bone formation while minimize adverse effects associated with high-level serum FGF2. We choose a β-globin promoter because 1) human *HB* , a specific gene encoding the beta chain of hemoglobin in erythroid cells, is strongly activated during the terminal stages of erythrocyte development [15]; 2) niches for erythropoiesis have been identified only in adult BM, and erythropoiesis is not spatially restricted to the areas proximal to sinusoids but occurs over the entire marrow space [16]; and 3) the β-globin promoter has been successfully used in stem cell gene therapy for treating red blood cell (RBC) diseases [17], [18]. In this study, we tested whether the use of the β-globin promoter can confine FGF2 expression to the marrow, thereby eliminating the adverse effects of high-level serum FGF2.

## Results

### FGF2 driven by the β-globin promoter confines FGF2 expression to the marrow cavity

**Figure 1:**
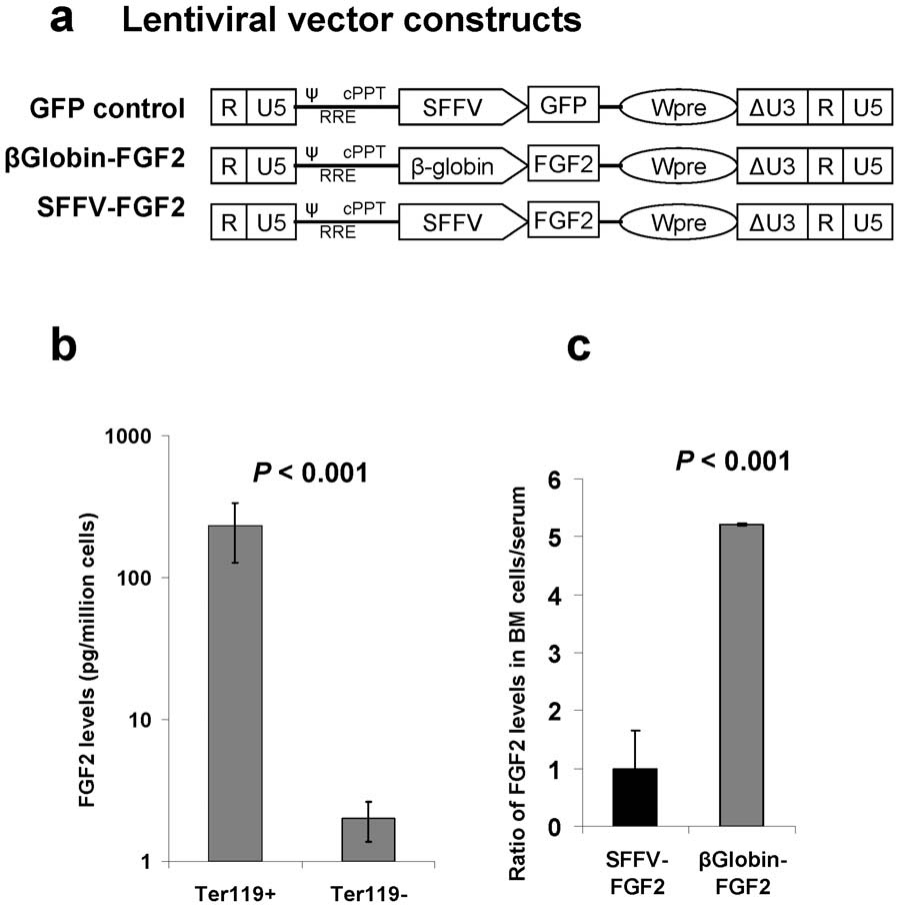
Use of the β-globin promoter confines FGF2 expression to the bone marrow. (a) Schematic of the self-inactivating (SIN) lentiviral vector for expression of the modified human FGF2 and GFP control. Δ indicates the SIN design with partially deleted U3 of the 3′ long terminal repeat. cPPT, central polypurine tract; Wpre, posttranscriptional regulatory element; RRE, rev-responsive element; SFFV, spleen focus-forming virus U3 promoter; ψ, packaging signal; β-globin: the human β-globin promoter from 5′ translated region (1.6 kb). (b) ELISA analysis of FGF2 in BM erythroid (Ter119^+^) and non-erythroid (Ter119^−^) cells demonstrates the specificity of the β-globin promoter. (**c**) The use of β-globin promoter leads to confined expression of FGF2 to the marrow. Serum levels and FGF2 expression were determined in marrow cells from mice that had received Sca-1^+^ cells transduced with Lenti β-globin-FGF2 or Lenti SFFV-FGF2 (n = 5 mice/group). The relative ratio of FGF2 levels in serum to the marrow is presented.

In a previous study, we used MLV, a gammaretroviral vector, to express FGF2 [13]. Here we used a lentiviral vector (Lenti) to express transgene, because self-inactivating lentiviral vectors are less genotoxic than gammaretroviral vectors due to their differential preference for integration [19], [20]. To achieve high-level transgene expression in hematopoietic cells, we used a spleen focus-forming virus (SFFV) promoter as a ubiquitous promoter to drive FGF2 expression [21]. To confine transgene expression to the marrow, we used an erythroid lineage-specific promoter, β-globin (**Fig. 1a**). As a negative control, a green fluorescent protein (GFP)-expressing vector was used in our study (**Fig. 1a**).

We first tested the specificity of the β-globin promoter. Sca-1^+^ cells purified by magnetic cell sorting (MACS) were transduced with Lenti β-globin-FGF2 and transplanted into lethally irradiated C57BL/6 mice. Six weeks after transplantation, BM cells were harvested and separated into 2 populations: Ter119^+^ erythroid cells and Ter119^−^ cells. Enzyme-linked immunosorbent assay (ELISA) analysis of extracted cellular proteins showed that FGF2 expression in Ter119^+^ erythroid cells was more than 100-fold higher than in control Ter119^−^ cells (**Fig. 1b**). This result demonstrates that the use of the β-globin promoter can achieve highly specific expression of the transgene in BM erythroid cells. Of interest, Ter119^+^ cells in the spleen expressed FGF2 at 20–50 fold lower levels compared to BM Ter119^+^ cells and FGF2 was undetectable in mature red blood cells (not shown).

We further tested the hypothesis that erythroid cell-mediated FGF2 expression allows for the confining of FGF2 to the marrow because BM is the only identified niche for erythropoiesis. For this purpose, mice were transplanted with Sca-1^+^ cells that were transduced with either Lenti β-globin-FGF2 or Lenti SFFV-FGF2. Six weeks after transplantation, serum and BM cells were harvested for analysis of FGF2 by ELISA. To measure the confinement levels, we determined the relative ratio of marrow FGF2 to serum FGF2. FGF2 expression in marrow cells was used as a surrogate for local FGF2 levels in the marrow. As expected, this ratio was increased by 5-fold in the β-globin-FGF2 animals compared to the SFFV-FGF2 animals (**Fig. 1c**). This result demonstrates that the β-globin promoter-mediated FGF2 expression in erythroid cells leads to a 5-fold enrichment of FGF2 in the marrow cavity relative to serum.

### Confined FGF2 expression leads to greater bone formation than SFFV-mediated ubiquitous FGF2 expression after myeloablative HSC transplantation

In our pilot studies, we observed that ∼20% of the mice could not survive to 6 weeks after myeloablative HSC transplantation due to side effects like severe anemia. We hypothesized that the severe side effect in the SFFV-FGF2 group might be due to the high-level expression of FGF2 driven by a strong ubiquitous promoter. To test this, we diluted Lenti SFFV-FGF2 transduced cells with untransduced Sca-1^+^ cells by factors of 1, 2, 4 and 8 before transplantation. At 6–8 weeks after transplantation, we sacrificed animals and analyzed serum FGF2 and parameters of bone formation.

**Figure 2:**
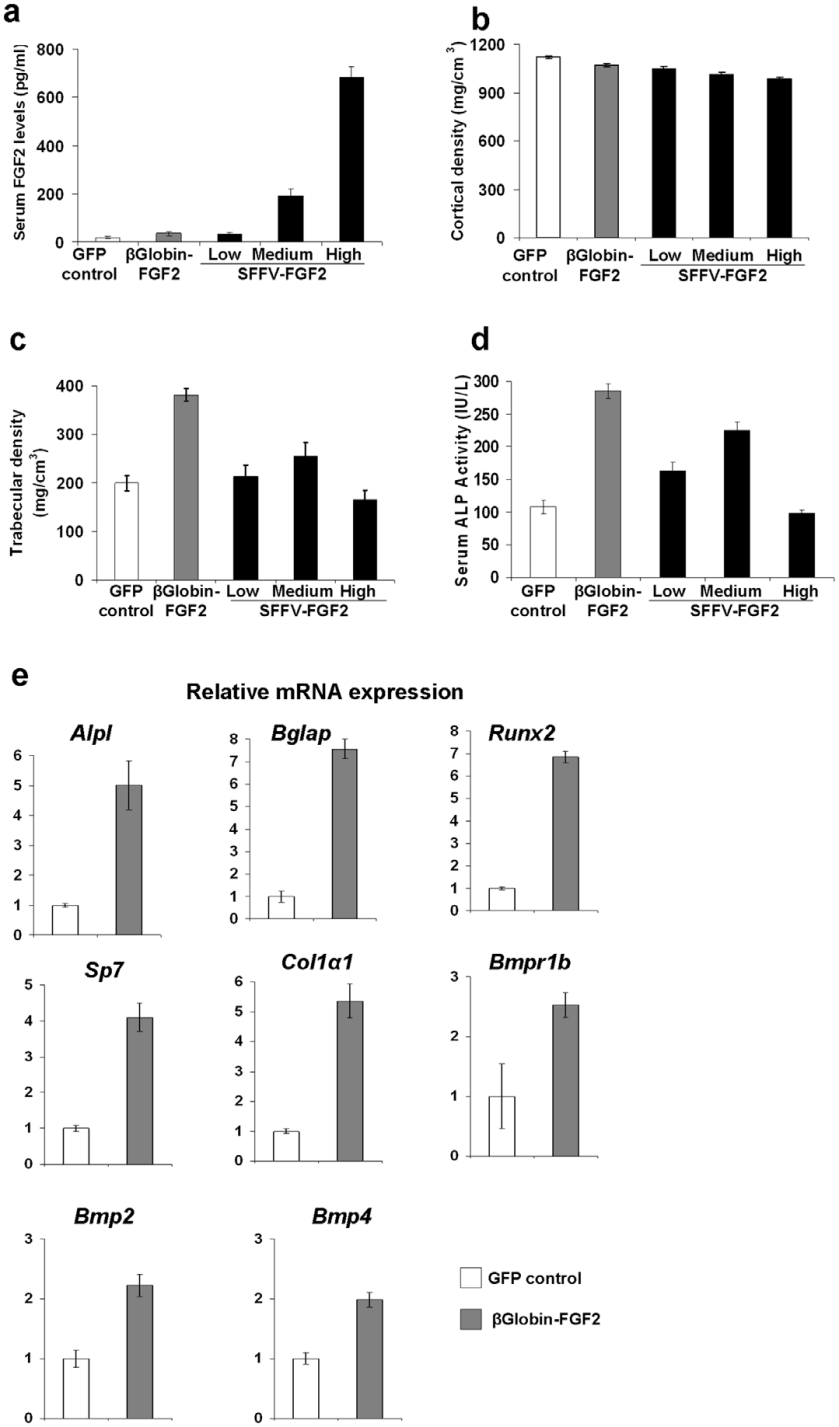
Confined expression of FGF2 in the marrow substantially increases bone formation. (a) Serum FGF2 levels in different groups of animals. To analyze the FGF2 dosage effects, the SFFV-FGF2 mice were stratified into 3 groups. (b) Cortical bone mineral density (BMD) as determined by pQCT analysis. GFP control vs. β-globin-FGF2 or low SFFV-FGF2, not significant; GFP control vs. medium SFFV-FGF2, *P*<0.05; GFP control vs. high SFFV-FGF2, *P*<0.01. (c) Trabecular bone mineral density (BMD) as determined by pQCT analysis. GFP control vs. β-globin-FGF2, *P*<0.001; GFP control vs. low SFFV-FGF2, not significant; GFP control vs. medium SFFV-FGF2, *P*<0.01. (d) Serum ALP activity at ∼8 weeks after transplantation. GFP control vs. β-globin-FGF2, *P*<0.001; GFP control vs. low SFFV-FGF2, *P*<0.001; GFP control vs. medium SFFV-FGF2, *P*<0.01; β-globin-FGF2 vs. low SFFV-FGF2, *P*<0.001; β-globin-FGF2 vs. medium SFFV-FGF2, *P*<0.001. (**e**) Real-time RT-PCR analysis of osteogenesis-related genes in the femurs. Alkaline phosphatase (*Alpl*), osteocalcin (*Bglap*), *Runx2*, osterix (*Sp7*), and collagen type Iα1 (*Col1a1*), bone morphogenetic protein receptor-1b (*Bmpr1b*), bone morphogenetic protein 2 (*Bmp2*), bone morphogenetic protein 4 (*Bmp4*), were analyzed. Data shown are presented as mean ± SEM (n = 10 mice/group).

To analyze effects of different FGF2 serum concentrations on bone formation, we grouped mice based on their serum FGF2 levels: 1) low levels (<100 pg/ml; range 11–80 pg/ml; mean 32 pg/ml), 2) medium levels (100–400 pg/ml; range 100–350 pg/ml; mean 190 pg/ml), and 3) high levels (>400 pg/ml; range 480–800 pg/ml; mean 680 pg/ml) (**Fig. 2a**). By comparison, average serum FGF2 levels in the GFP control group and the β-globin group were 17 pg/ml (range: 0–60 pg/ml) and 34 pg/ml (range: 1–80 pg/ml), respectively (**Fig. 2a**).

We conducted peripheral quantitative computed tomography (pQCT) analysis to determine the effects of FGF2 on bone formation. We observed a trend of decrease in cortical bone mineral density (BMD) with the increase of serum FGF2 (**Fig. 2b**). For trabecular bone formation, we observed that femurs from the β-globin group showed a substantial increase in BMD relative to the GFP control (*P*<0.001) (**Fig. 2c**). However, in the SFFV-FGF2 groups, only medium-level serum FGF2 induced a significant increase in trabecular BMD compared to the GFP control, while no significant differences were observed in the low and high serum GFP groups. Other bone parameters such as trabecular content measured by pQCT also showed the same pattern (data not shown). Of interest, there was no difference in serum FGF2 levels between the β-globin and the low SFFV-FGF2 group, but substantial trabecular bone formation was observed in the β-globin group (**Fig. 2c**). These data suggest that confined expression of FGF2 to the marrow leads to striking increase in trabeculae bone formation in endosteum.

We also measured the serum levels of alkaline phosphatase (ALP), a serum biomarker of bone formation. Consistent with the pQCT data, ALP activity at 6–8 weeks after transplantation was increased by ∼150% in the β-globin animals compared to the GFP control (**Fig. 2d**). In the SFFV-FGF2 mice, an increase of serum FGF2 from ∼30 pg/ml to ∼200 pg/ml significantly increased the ALP activity, while a further increase to ∼600 pg/ml led to a drop in the ALP activity (**Fig. 2d**). We also examined another serum biomarker of bone formation, osteocalcin, and saw the same pattern as the ALP activity (data not shown). These data further support our conclusion that β-globin promoter-mediated confined FGF2 expression leads to substantial bone formation.

To investigate the mechanisms of confined FGF2 expression-mediated bone formation, we extracted total RNAs from whole femurs and conducted real-time reverse transcription polymerase chain reaction (RT-PCR) analysis. The GFP group served as a negative control. Consistent with the serum ALP (encoded by *Alp1*) and osteocalcin (encoded by *Bglap*) data, RT-PCR analysis showed that expression of *Alp1* and *Bglap* was increased ∼5 and ∼7-fold, respectively (**Fig. 2e**). Expression of *Runx2 and Sp7* (also known as *osterix*), transcription factors specifically expressed in osteoblasts, was increased 4–6 fold in the β-globin-FGF2 group relative to control (**Fig. 2e**), suggesting that FGF2 may promote osteoblast proliferation. Similarly, we observed that confined FGF2 expression led to a 2-fold increase in *Bmpr1b*, *Bmp2* and *Bmp4* (**Fig. 2e**), which is consistent with the report that FGF2 activates BMP signaling [22]. These data suggest that FGF2 promotes bone formation due in part to activation of BMP signaling. In addition, the expression of *Col1a1*, the gene that encodes the major extracellular matrix protein collagen 1a type 1, was increased by 5-fold in the β-globin-FGF2 group (**Fig. 2e**). This observation suggests that FGF2 also stimulates secretion of extracellular matrix. In the SFFV-FGF2 animals, the increase in expression of these genes was less pronounced than the β-globin-FGF2 animals (not shown).

Taken together, our data demonstrate that the β-globin promoter mediated confined FGF2 expression to the BM cavity substantially increases bone formation, likely by stimulating osteoblast proliferation and secretion of osteogenic factors like Bmp2 and Bmp4.

### Confined FGF2 expression eliminates high-level FGF2 associated anemia and ameliorates extramedullary hematopoiesis

**Figure 3:**
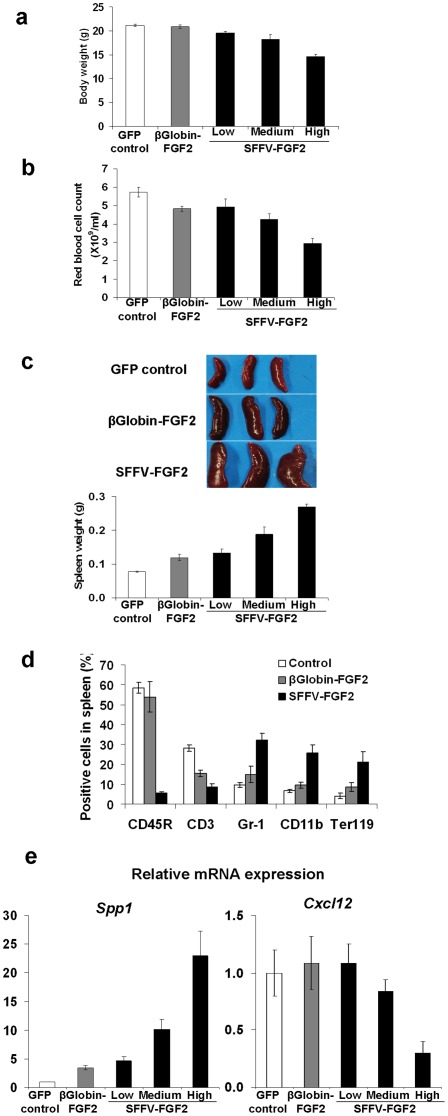
Confined FGF2 expression eliminates high-level FGF2 associated anemia and ameliorates extramedullary hematopoiesis. (a) FGF2 negatively affects body weight. Mice were weighed at 8 weeks after transplantation. GFP control vs. β-globin-FGF2, not significant; GFP control vs. low SFFV-FGF2, *P*<0.05; GFP control vs. medium SFFV-FGF2, *P*<0.001; GFP control vs. high SFFV-FGF2, *P*<0.001. (b) FGF2 negatively affects red blood cell count. Red blood cells in peripheral blood were counted at 8 weeks after transplantation. GFP control vs. β-globin-FGF2, *P*<0.05; GFP control vs. low SFFV-FGF2, *P*<0.05; GFP control vs. medium SFFV-FGF2, *P*<0.01; GFP control vs. high SFFV-FGF2, *P*<0.001. (c) FGF2 induces extramedullary hematopoiesis in a concentration-dependant manner. Shown are representative spleens and the spleen weight in different groups. GFP control vs. β-globin-FGF2, *P*<0.05; GFP control vs. low SFFV-FGF2, *P*<0.05; GFP control vs. medium SFFV-FGF2, *P*<0.01; GFP control vs. high SFFV-FGF2, *P*<0.001. (d) Flow cytometry analysis of CD45R^+^ B cells, CD3^+^ T cells, Gr-1^+^ and CD11b^+^ myeloid cells, and Ter119^+^ erythroid cells in spleens from the GFP control mice, the β-globin-FGF2 mice and the high SFFV-FGF2 mice at 8 weeks after transplantation. (e) Relative mRNA expression of *Spp1*, and *Cxcl12* in femurs from the GFP control mice, the β-globin-FGF2 mice and the high SFFV-FGF2 mice. For *Spp1*, GFP control vs. all the other groups, *P*<0.001. For *Cxcl12*, GFP control vs. high SFFV-FGF2, *P*<0.001; others, GFP control vs. other groups, not significant. Data shown are presented as mean ± SEM (n = 10 mice/group).

Systemic FGF2 administration induces side effects such as extramedullary hematopoiesis and anemia [23]. Therefore, we hypothesized that the anemia might be induced by high-level serum FGF. Gross examination of the animals showed that body weight in the SFFV-FGF2 animals was lower than control in a FGF2 concentration-dependent manner, with high-level serum FGF2 leading to a 20% decrease at 8 weeks after transplantation (*P*<0.001) (**Fig. 3a**). This observation is consistent with the earlier report that FGF2 inhibits weight gain in growing animals [24]. To investigate the effects of FGF2 on anemia, we counted RBC in peripheral blood. **Fig. 3b** shows that even low-level serum FGF2 decreased RBC count by 10–15% (*P*<0.05), while further increase in FGF2 led to an additional drop in the RBC count. In the high SFFV-FGF2 group, the RBC counts were decreased by ∼40% relative to control (*P*<0.001), leading to severe anemia. The observed anemia in animals with high serum FGF2 levels may have contributed to the retarded body weight gain and sickness in some animals. In the β-globin-FGF2 animals, the RBC counts were also decreased to the same level as in the low SFFV-FGF2 animals (**Fig. 3b**). These data suggest that FGF2 plays a negative role in erythropoiesis in a serum FGF2 dose-dependent manner, and the β-globin promoter mediated confined FGF2 expression decreases serum FGF2 levels and thereby prevents anemia. Of interest, no significant difference in white blood cell counts between all the groups was observed, while platelet counts were significantly decreased in mice with medium- or high-level serum FGF2 (not shown).

We and others have shown that FGF2 induces extramedullary hematopoiesis [13]. As expected, we also observed that FGF2 serum levels were positively associated with the spleen weight (**Fig. 3c**). To analyze cell subpopulations in the spleen, we minced the spleens and analyzed cells by flow cytometry. In the high SFFV-FGF2 group, the portion of the CD45R^+^ B cells decreased by 10-fold and the CD3^+^ T cells decreased by ∼3-fold, while the Gr-1^+^ or CD11b^+^ myeloid cells and the Ter119^+^ erythroid cells increased by 3–5 fold relative to control (**Fig. 3d**). These results are suggestive of extramedullary hematopoiesis. The decreased percentages of B and T cells in the spleens can be explained by the dilution effect of strikingly increased myeloid and erythroid cells in the high SFFV-FGF2 animals. We also observed a significant increase in percentage of LSK (Lin^−^Sca1^+^c-kit^+^) stem cells in the spleen of mice with high FGF2, suggesting stem cell mobilization. By contrast, the change in the subpopulation profile was less pronounced in the β-globin-FGF2 animals (**Fig. 3d**). These data suggest that the β-globin promoter mediated confined FGF2 expression leads to less pronounced extramedullary hematopoiesis.

We speculated that FGF2-associated extramedullary hematopoiesis and anemia is due to stem cell dysregulation. To test this hypothesis, we analyzed expression of genes associated with stem cell regulation in BM cells by real-time RT-PCR. We found that expression of *Spp1*, which encodes osteopontin, increased substantially in an FGF2 dose-dependent manner (*P*<0.001) (**Fig. 3e**). Given that osteopontin is a negative regulatory factor in the stem cell niche that controls the stem cell number [25], this result suggests that FGF2 decreases the stem cell pool in the marrow by upregulating osteopontin. High-level FGF2 also led to an approximately 70% decrease in *Cxcr12* (also known as *SDF-1*) expression (*P*<0.001) (**Fig. 3e**), which is consistent with an earlier report that FGF2 posttranscriptionally down-regulates *SDF-1* expression [26]. Because SDF-1/CXCR4 axis modulates homing, BM retention and mobilization of HSCs, a marked decrease of *SDF-1* in the high SFFV-FGF2 group may have led to substantial stem cell mobilization, contributing to splenomegaly (**Fig. 3c**). In contrast, in the β-globin-FGF2 animals, the increase of *Spp1* expression was less pronounced and no significant change in *CXCR12* was observed (**Fig. 3e**). These data suggest that high-level FGF2 may decrease HSC pool by negatively regulating stem cell niches and decrease marrow cellularity by attenuating BM retention capacity, while confined FGF2 expression substantially ameliorates these effects and thus prevents severe anemia.

### Confined FGF2 expression eliminates high-level FGF2-associated osteomalacia

**Figure 4:**
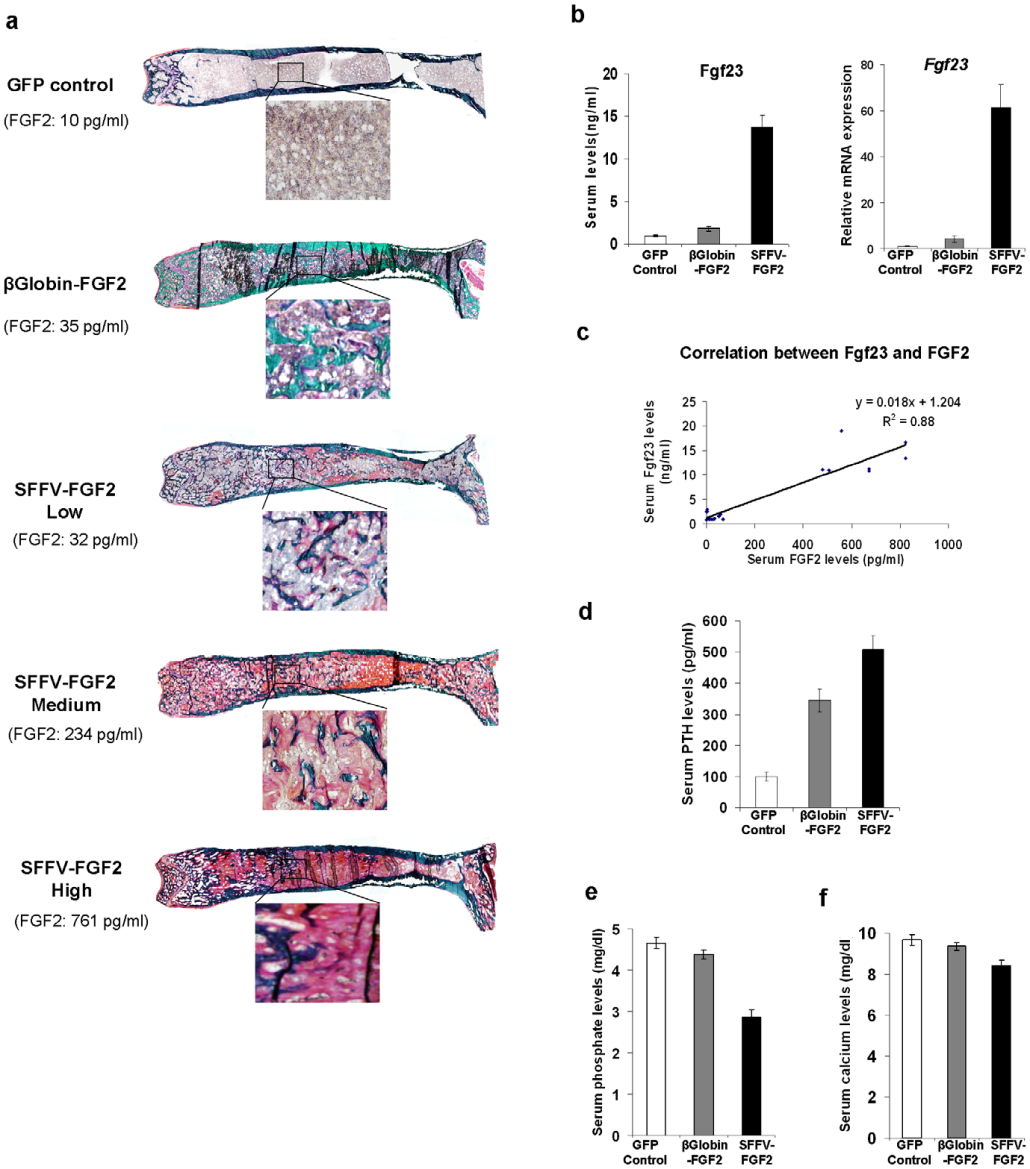
Confined FGF2 expression abrogates high-level FGF2-associated osteomalacia. (a) Representative illustration of Goldner's trichrome staining of femurs from each group. Green indicates mineralized bone and red or pink unmineralized bone. (b) Serum Fgf23 levels and relative *Fgf23* mRNA expression in femurs from the GFP control mice, the β-globin-FGF2 mice and the high SFFV-FGF2 mice. *P*<0.001 for all comparisons. (c) Serum Fgf23 is positively correlated with serum FGF2 levels. *P*<0.001. (d). Serum levels of PTH. *P*<0.001 for all comparisons. (e) Serum phosphate levels. GFP control vs. β-globin-FGF2, *P* = 0.06; *P*<0.001 for all the other comparisons. (f) Serum calcium levels. GFP control vs. β-globin-FGF2, *P* = 0.40; *P*<0.01 for all the other comparisons. Data shown are presented as mean ± SEM (n = 10 mice/group).

FGF2 administration impairs bone mineralization [27] and high-level serum FGF2 leads to osteomalacia [13]. We thus conducted Goldner's trichrome staining on femurs to examine bone minerization. As expected, in the SFFV-FGF2 group, we observed unmineralized bone and the areas of unmineralized bone were positively associated with serum FGF2 levels (**Fig. 4a**). In sharp contrast, the newly formed bone was completely mineralized in the β-globin-FGF2 animals (**Fig. 4a**). These data demonstrate that confined FGF2 expression abrogates osteomalacia that is associated with high-level serum FGF2 levels. Of interest, even in the low SFFV-FGF2 group, in which the same FGF2 levels were detected as in the β-globin group, areas of unmineralized bone were still observed (**Fig. 4a**).

We further investigated the mechanisms underlying complete minerization in the β-globin-FGF2 animals but development of osteomalacia in the SFFV-FGF2 animals. We hypothesized that FGF23 may be a crucial factor because previous studies show that FGF23 is a causative factor of hypophosphatemia and osteomalacia [28], [29] and forced *FGF23* expression in osteoblasts inhibits mineralization and promotes osteomalacia [30]. To test our hypothesis, we first examined serum Fgf23 levels by ELISA. The serum Fgf23 levels in the high SFFV-FGF2 animals increased more than 13-fold (*P*<0.001), while Fgf23 was only slightly increased in the β-globin-FGF2 animals (**Fig. 4b**). Consistent with this result, real-time RT-PCR analysis of femurs indicated a more than 80-fold upregulation of *Fgf23* in the high SFFV-FGF2 group (*P*<0.001), while the increase of *Fgf23* expression in the β-globin-FGF2 group was ∼5-fold (**Fig. 4b**). Furthermore, regression analysis showed a linear positive association between serum FGF2 and Fgf23 (*P*<0.001) (**Fig. 4c**). These data suggest that attenuated Fgf23 levels in animals with confined FGF2 expression has contributed to the abrogation of osteomalacia.

Secondary hyperparathyroidism is often associated with osteomalacia [31]; we thus measured serum levels of PTH. As expected, in the high SFFV-FGF2 animals, PTH was increased by 5-fold (*P*<0.001), while the increase in the β-globin-FGF2 animals was less pronounced (*P*<0.01) (**Fig. 4d**). FGF23 regulates phosphate homeostasis and high-level FGF23 leads to depletion of serum phosphates [31]. As expected, in the high SFFV-FGF2 animals, the phosphate levels were substantially lower than control (2.8 mg/dl vs. 4.7 mg/dl; *P*<0.001), while only a slight decrease of serum phosphate in the β-globin-FGF2 animals was observed (*P* = 0.06) (**Fig. 4e**). In addition, consistent with our previous study, we also observed hypocalcaemia in high SFFV-FGF2 animals (8.5±0.2 mg/dl vs. 9.7±0.3 mg/dl for control; *P*<0.01) (**Fig. 4f**). However, the calcium levels in the β-globin-FGF2 animals were in the normal range (9.4±0.2 mg/dl; *P* = 0.40) (**Fig. 4f**).

Taken together, our data suggest that high-level Fgf23, hypophosphatemia and hypocalcaemia may have contributed to secondary hyperparathyroidism and osteomalacia in the SFFV-FGF2 animals, while low-level Fgf23 in the β-globin-FGF2 animals does not lead to a significant depletion of phosphate, thereby preventing osteomalacia.

### High-level serum FGF2 induces considerable trabecular bone formation after nonmyeloablative HSC transplantation but still promotes anemia and osteomalacia

**Figure 5:**
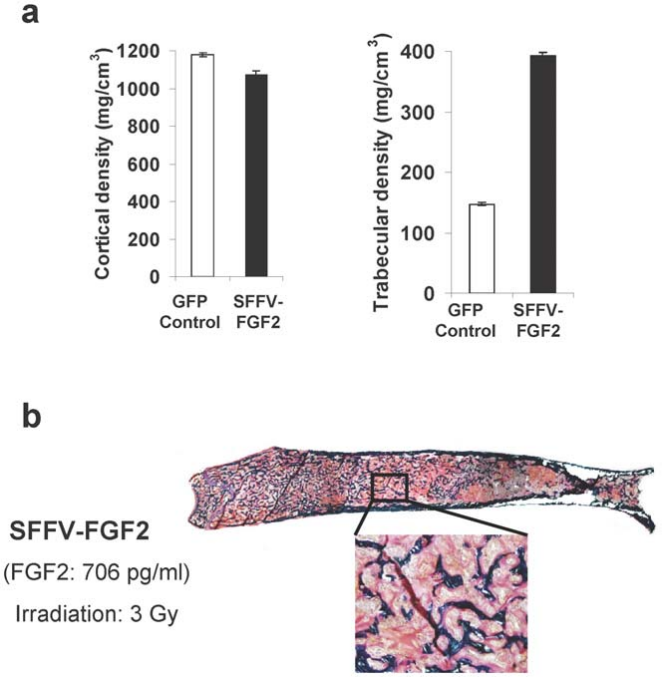
High-level FGF2 induces considerable trabecular bone formation after nonmyeloablative HSC transplantation but develops osteomalacia. (a) pQCT analysis of cortical and trabecular bone mineral density at 11 weeks after transplantation. Substantial trabecular bone formation was detected. For trabecular bone, *P*<0.001. Animals received GFP control or lenti SFFV-FGF2 transduced cells after 3 Gy irradiation. (b) Goldner's trichrome staining of a femur from a representative SFFV-FGF2 mouse at 11 weeks after transplantation. Green colored is mineralized bone. Red colored tissue indicates unmineralized bone area.

In the above studies, we observed less pronounced trabecular bone formation and obvious animal illness in the high SFFV-FGF2 group, which is apparently inconsistent with our previous report in which a sublethal irradiation dosage was used [13]. We reasoned that this might be because lethally irradiated animals are more susceptible to high-level FGF2-associated adverse effects like anemia, leading to severe sickness at ∼6 weeks after transplantation. To test our hypothesis that irradiation dosage is a crucial factor underlying the discrepancy, we irradiated animals at 3 Gray (Gy) before HSC transplantation. To ensure HSC engraftment in a non-myeloablative transplantation setting, we expanded cells with a homeobox (HOX) gene [32] after transduction of Sca-1^+^ cells with Lenti SFFV-FGF2. One week after transduction, 1×10^7^ Sca1^+^ cells were transplanted into each mouse. At 11 weeks after transplantation, we euthanized the animals for analysis. We observed high-level FGF2 in these animals (∼600 pg/ml). While the cortical BMD was slightly decreased in the SFFV-FGF2 animals, the trabecular BMD was markedly increased from ∼150 mg/cm^3^ in control animals to ∼400 mg/cm^3^ in high FGF2 animals (**Fig. 5a**). This result is similar to the considerable trabecular bone formation observed in the β-globin-FGF2 animals (**Fig. 2b**). Godner's trichrome staining also confirmed the effects of high FGF2 on bone formation (**Fig. 5b**). However, the overwhelming osteomalacia was still evident (**Fig. 5b**). In addition, we observed overt hypophosphatemia in all the animals and anemia in some (data not shown). Together, this study resolved the apparent discrepancy and consolidated the conclusion that the β-globin promoter mediated confined FGF2 expression attenuates anemia and eliminates osteomalacia.

## Discussion

Here we report that use of the β-globin promoter leads to a 5-fold enrichment of FGF2 expression in the marrow compared to the ubiquitous promoter SFFV-mediated transgene expression. The confined expression of FGF2 leads to a substantial increase in trabecular bone formation with no severe side effects like anemia and osteomalacia that are associated with systemic administration of FGF2. In contrast to our earlier report, the new approach leads to considerable trabecular bone formation with only slightly increased serum FGF2 relative to baseline levels of ∼10 pg/ml. The decrease in serum FGF2 levels from ∼1000 pg/ml to ∼30 pg/ml may also eliminate the potential untoward effects of high-level FGF2, such as promoting tumorigenesis in concert with other factors.

FGF2 remarkably and rapidly enhances bone formation through its stimulatory effects on proliferation [33], [34], [35] and survival [36] of osteoprogenitor cells. However, after two decades of development, systemic FGF2 administration has not been used in clinical therapy to enhance osteogenesis, largely due to its severe side effects [13], [27]. To minimize the adverse effects of high-level FGF2, we proposed a new concept of confining FGF2 to the marrow and thus promoting trabecular bone formation by using an erythroid-specific promoter to drive FGF2 expression. Because marrow is the only identified niche for erythropoiesis, the β-globin promoter-mediated FGF2 expression leads to considerable trabecular bone formation and substantially decreases serum FGF2 to levels that are only slightly higher than physiological concentrations. Our finding has important implications for the safety of FGF2-based therapy. FGF2 is a pleiotropic factor that affects the growth, differentiation, and survival of various cell types. Its overexpression promotes tumor neovascularization and metastasis [37] and aberrant FGF signaling is also associated with cancer development and progression [38]. Our advance in decreasing serum FGF2 levels by 5-fold with the use of the β-globin promoter might largely eliminate this concern.

In addition to minimizing the safety concern of systemic FGF2 therapy, our new approach for HSC gene therapy is also safer to use in clinical applications than previous strategies. First, self-inactivating lentiviral vectors are apparently safer than gammaretroviral vectors in terms of genotoxicity and oncogenic potential, in particular when the enhancer element is depleted from the vector [19], [20]. Second, in contrast to viral promoters, no silencing effects of lineage-specific promoter have been observed, which makes it easier to control the dosage of therapeutic genes. Third, the use of lineage-specific promoter in HSC gene therapy enhances safety [39]. Although uncontrolled activation of growth-promoting genes in stem cells can lead to oncogenesis, this is unlikely if vector transcriptional activity is restricted to fully differentiated cells [18], [40].

In our high SFFV-FGF2 group, we reproduced high-level FGF2-induced adverse effects such as anemia and osteomalacia. Our mechanistic analyses suggest that these are induced by FGF2's effects on hematopoiesis and phosphate homeostasis. Earlier studies suggest that FGF2 negatively affects normal BM hematopoiesis by several mechanisms: 1) destroys the CXCL12/CXCR4 axis by downregulation of CXCL12, leading to stem cell mobilization [26]; and 2) limits the size of the stem cell pool by increasing osteopontin (*Spp1*) expression [25]. Our analysis of femurs from the high SFFV-FGF2 group confirmed these findings. FGF2-mediated decrease of stem cell pool and mobilization of stem cells out of marrow niche result in dysfunctional marrow hematopoiesis and compensatory extramedullary hematopoiesis. However, even with massive (∼20-fold) increase in erythroid cells in the spleen, the inefficient erythropoiesis outside of erythroblast islands leads to anemia. In contrast to animals with high-level FGF2, the β-globin promoter-mediated confined FGF2 expression substantially ameliorates the negative effects of FGF2 on hematopoiesis, and development of overt anemia was not observed in these animals.

The second major unintended effect of high-level FGF2 is osteomalacia. Our data and related publications demonstrate that FGF23 plays a pivotal role in osteomalacia-related side effects [31], [41]. FGF23 is a phosphaturic hormone that acts on the kidney to increase phosphate excretion; high-level FGF23 results in depletion of phosphate from the body, leading to osteomalacia. We observed a strong correlation between Fgf23 levels and PTH levels and unmineralized bone areas. Increased Fgf23 production may have contributed to the development of secondary hyperparathyroidism [42]. An alternative explanation is that high demand for calcium due to massive bone formation induces hypocalcaemia, which gives rise to secondary hyperparathyroidism, which induces upregulation of Fgf23 and depletion of phosphate leading to osteomalacia. However, this inference is not supported by our observation in the β-globin FGF2 animals, in which considerable trabecular bone formation was observed, while these animals manifest less severe hyperparathyroidism and hypophosphatemia compared to high SFFV-FGF2 animals. Therefore, our data suggest that hypocalcemia and hypophosphatemia are a consequence rather than a cause of high-level Fgf23 in the SFFV-FGF2 animals.

The causative link between FGF2 and Fgf23 is still missing. We observed a strong positive correlation between serum FGF2 and serum Fgf23, but whether FGF2 directly upregulates Fgf23 is unknown. A recent report shows that overexpression of nuclear high molecular weight FGF2 isoform increases FGF23/FGFR/KLOTHO signaling, causing phosphate wasting and osteomalacia [43]. Similarly, another study demonstrates that FGFR signaling stimulates Fgf23 expression in a dose-dependent manner [44]. These findings suggest that overexpression of FGF2 is a contributing factor to FGF23 upregulation in our animals.

We found that the use of β-globin promoter leads to an impressive confinement of FGF2 expression to the marrow. To the best of our knowledge, we are the first to report that an erythroid promoter can be used to drive the expression of therapeutic genes to the marrow after HSC transplantation. This finding should have important implications in delivering therapeutic factors to the marrow for treating hematological diseases such as leukemia.

In the previous study, we found that high-level FGF2 induces massive trabecular formation [13], which is seemingly inconsistent with our study with myeloablative transplantation. We reasoned that the discrepancy might be attributed to the different irradiation dosage we used: 5 Gy for the earlier study vs. 8 Gy in the majority of our current study. To test our hypothesis, we provided evidence that in non-myeloablative transplantation, high-level FGF2 can induce substantial trabecular bone formation but also induce overt osteomalacia.

In conclusion, our improved hematopoietic stem cell gene therapy approach has advanced FGF2-based therapy for bone regeneration in multiple ways. The use of an erythroid-specific promoter confines FGF2 expression to the marrow, leading to a substantial decrease in serum FGF2. Our new strategy not only promotes massive trabecular bone formation, but also leads to abrogation of the side effects associated with systemic FGF2 administration such as anemia and osteomalacia. Our improved therapy represents one step closer to clinical applications in strengthening the skeleton and battling osteoporosis. At the same time, we are aware that toxic irradiation used in HSC transplantation as a preconditioning regimen can not be used in non-life-threatening diseases like osteoporosis, and we are addressing this issue by exploiting integrin and SDF-1/CXCR4 signaling pathways (Meng, Baylink, Lau, and Zhang: unpublished).

## Materials and Methods

### Lentiviral vectors

We used lentiviral vectors to express a modified human FGF2 with a BMP2/4 hybrid secretion signal [45]. In this study, GFP control vector and 2 FGF2-expressing vectors were used, in which SFFV, a strong promoter for hematopoietic cells [21], [46], and β-globin promoter, an erythroid-specific promoter of 1.6 kb in length, were used to drive FGF2 expression [47] (**Fig. 1a**). For virus production, a standard calcium phosphate precipitation method was used. The biological titers of lentiviral vectors were determined with HT1080 cells (a human fibrosarcoma cell line). 0.1, 0.2 or 0.5 µl concentrated vectors were added into each well of 12-well plates that were seeded with 5×10^4^ cells. As a control, some wells of HT1080 cells were added with Lenti-PGK-GFP-wpre virus, whose titer was calculated by flow cytometry analysis of percentage of GFP^+^ cells. After 3 days of culture in DMEM/10% fetal bovine serum (Invitrogen, Grand Island, NY) with addition of 8 µg/ml protamine sulfate (Sigma-Aldrich Corp,, St. Louis, MO), cells were harvested for genomic DNA extraction using the DNeasy kit (Qiagen, Inc., Valencia, CA). To determine the average copies of integrated lentivirus, real-time PCR was performed using the SYBR® Green PCR Master Mix (Applied Biosystems, Foster City, CA) on 7500 Fast Real-Time PCR System (Applied Biosystems). The titers of non-GFP-expressing vectors were calculated by normalization with HT1080 cells transduced with Lenti-PGK-GFP-wpre. After a 100× concentration, biological titers of more than 5×10^7^/ml were achieved in our lab [21], [48].

### Mice

Six-week old female C57BL/6 mice were purchased from the Jackson Laboratory (Bar Harbor, ME). All animals were maintained in the Loma Linda University Animal Facility. All procedures were conducted under humane conditions and approved by the Institutional Animal Care and Use Committee at the Loma Linda University.

### Bone marrow Sca-1^+^ cells

Whole BM cells were harvested from C57BL/6 mice by flushing tibiae and femurs with phosphate-buffered saline (PBS) plus 0.1% bovine serum albumin using a 26G syringe. After depletion of red blood cells by treatment with RBC lysis buffer, nucleated cells were enriched for Sca-1^+^ cells by MACS using a Sca-1 Microbead kit from Miltenyi Biotec, Auburn, CA.

### Cell transduction

Sca-1^+^ cells were plated in non-tissue culture (TC) treated 6-well plates (Becton Dickinson, Franklin Lakes, NJ) pre-coated with retronectin (Takara, Otsu, Shiga, Japan) at a density of 2×10^6^ cells/well. Cells were cultured in Iscove's modified Dulbecco's medium (Invitrogen) containing 10% fetal bovine serum (Invitrogen), human TPO (Prospec, East Brunswick, NJ), mouse SCF (Prospec), human FL (Prospec), human G-CSF (Prospec), and human IL-3 (Prospec), each at 100 ng/ml. After overnight prestimulation, concentrated viral stock was added to the cell culture at a multiplicity of infection of 5. Eight hours later, cells were harvested and resuspended in PBS for transplantation.

### Mouse irradiation and transplantation

Recipient C57BL/6 mice were placed into aerated polystyrene cubicles and preconditioned by total body irradiation from a ^60^Co source (Eldorado model, Atomic Energy of Canada, Ltd., Commercial Products Division, Ottawa, Canada) at a single dose of 8 Gy; dose rate was approximately 0.8 Gy/minute. Two to three hours after irradiation, transduced 0.5×10^6^ Sca-1^+^ cells were transplanted into syngeneic C57BL/6 mice via tail vein injection. To investigate the effects of differential concentrations of FGF2 on bone formation, SFFV-FGF2 transduced cells were diluted with un-transduced Sca-1^+^ cells by factors of 2, 4 or 8 before transplantation.

### FGF2 expression in Ter119 positive and negative cells

To investigate the specificity of the β-globin promoter, we harvested BM cells from mice received Lenti β-globin-FGF2 transduced cells at 6 weeks after transplantation. Erythroid cells were purified by MACS with the use of an Anti-Ter119 Microbead kit (Miltenyi Biotec). Ter119^−^ cells were also collected as a negative control. Protein was extracted from isolated cells using Qproteome™ Nuclear Protein kit (Qiagen). FGF2 expression in Ter119^+^ or Ter119^−^ cells was determined by ELISA analysis of protein extracts.

### Serum analysis

Mice were sacrificed by CO_2_ asphyxiation at 6–8 weeks after transplantation and serum was harvested for a series of analyses. FGF2 levels were analyzed by ELISA (R&D Systems, Minneapolis, MN). Activity of bone formation markers, osteocalcin and ALP, was measured with osteocalcin ELISA kit (Biomedical Technologies Inc., MA) and Quantichrom Alkaline Phosphatase Assay Kit (BioAssay Systems, Hayward, CA), respectively. We also analyzed indicators of osteomalacia, PTH and FGF23, using PTH Immunoassay Kit (ALPCO, Salem, NH) and FGF23 ELISA kit (Immutopics International, San Clemente, CA), respectively.

### pQCT measurements

Femurs were fixed in 10% formalin for 24 hours and then stored in PBS containing 0.1% sodium azide at 4°C. Cross-sectional and volumetric bone parameters (trabecular and cortical BMD) were measured using an XCT 960M with XCT software version 5.40 (Roche Diagnostics, Basel, Switzerland) in a multi-specimen holder designed for the XCT 960M as previously described [13].

### Bone tissue histology

To examine the bone mineralization, femurs were embedded into methylmethacrylate. A Polycut motorized microtome was used to cut 5-µM-thick sections; the microsections were stained with Goldner's trichrome stain as previously described [49].

### Fluorescence-activated cell sorting

Spleen cells were obtained by mincing the spleen. After lysis of RBC, nucleated cells were washed in PBS and incubated for 30 minutes with phycoerythrin (PE)-conjugated mouse monoclonal antibodies. The antibodies were used to identify cells expressing CD45R, Gr-1, CD11b, CD3 and Ter119 (eBioscience, San Diego, CA). Nonspecific staining was excluded using a mouse isotope immunoglobulin control. 30,000 cells from each sample were analyzed on a FACSAria II flow cytometer (BD Biosciences, San Jose, CA).

### RT-PCR

Quantitative real-time reverse transcription PCR was performed to determine the gene expression of multiple markers in whole femurs. After harvesting and cleaning femurs, the samples were snap frozen in liquid nitrogen. To extract RNAs, the femurs were pulverized. Total RNA was isolated using TRIzol Reagent (Invitrogen). First strand cDNA was synthesized using SuperScript® III First-Strand Synthesis System and the OligodT primer (Invitrogen). Real-time PCR was performed using SYBR® Green PCR Master Mix (Applied Biosystems) on 7500 Fast Real-Time PCR System (Applied Biosystems). Gene expression values were normalized against glyceraldehyde 3-phosphate dehydrogenase (*Gapdh*), and relative quantitation of fold change was calculated using the 2^−ΔΔCT^ method. Primer sequences for RT-PCR are listed in Table S1 in Supporting Information S1.

### Statistical analysis

Data are presented as mean ± standard error of the mean (SEM). Two-tailed Student's *t* test was performed. *P* value of <0.05 was considered statistically significant.

## Supporting Information

Table S1 Primer sequences for real-time RT-PCR.
(TIF)

## References

[1] Anderson GL, Limacher M, Assaf AR, Bassford T, Beresford SA (2004). Effects of conjugated equine estrogen in postmenopausal women with hysterectomy: the Women's Health Initiative randomized controlled trial.. JAMA.

[2] Lewiecki EM (2010). Treatment of osteoporosis with denosumab.. Maturitas.

[3] Ominsky MS, Vlasseros F, Jolette J, Smith SY, Stouch B (2010). Two doses of sclerostin antibody in cynomolgus monkeys increases bone formation, bone mineral density, and bone strength.. J Bone Miner Res.

[4] Wang JS (1996). Basic fibroblast growth factor for stimulation of bone formation in osteoinductive or conductive implants.. Acta Orthop Scand.

[5] Nakamura K, Kawaguchi H, Aoyama I, Hanada K, Hiyama Y (1997). Stimulation of bone formation by intraosseous application of recombinant basic fibroblast growth factor in normal and ovariectomized rabbits.. J Orthop Res.

[6] Nakamura T, Hanada K, Tamura M, Shibanushi T, Nigi H (1995). Stimulation of endosteal bone formation by systemic injections of recombinant basic fibroblast growth factor in rats.. Endocrinology.

[7] Lane NE, Kumer J, Yao W, Breunig T, Wronski T (2003). Basic fibroblast growth factor forms new trabeculae that physically connect with pre-existing trabeculae, and this new bone is maintained with an anti-resorptive agent and enhanced with an anabolic agent in an osteopenic rat model.. Osteoporos Int.

[8] Iwaniec UT, Magee KA, Mitova-Caneva NG, Wronski TJ (2003). Bone anabolic effects of subcutaneous treatment with basic fibroblast growth factor alone and in combination with estrogen in osteopenic ovariectomized rats.. Bone.

[9] Power RA, Iwaniec UT, Wronski TJ (2002). Changes in gene expression associated with the bone anabolic effects of basic fibroblast growth factor in aged ovariectomized rats.. Bone.

[10] Yao W, Hadi T, Jiang Y, Lotz J, Wronski TJ (2005). Basic fibroblast growth factor improves trabecular bone connectivity and bone strength in the lumbar vertebral body of osteopenic rats.. Osteoporos Int.

[11] Montero A, Okada Y, Tomita M, Ito M, Tsurukami H (2000). Disruption of the fibroblast growth factor-2 gene results in decreased bone mass and bone formation.. J Clin Invest.

[12] Lane NE, Yao W, Kinney JH, Modin G, Balooch M (2003). Both hPTH(1–34) and bFGF increase trabecular bone mass in osteopenic rats but they have different effects on trabecular bone architecture.. J Bone Miner Res.

[13] Hall SL, Lau KH, Chen ST, Wergedal JE, Srivastava A (2007). Sca-1(+) hematopoietic cell-based gene therapy with a modified FGF-2 increased endosteal/trabecular bone formation in mice.. Mol Ther.

[14] Hall SL, Chen ST, Gysin R, Gridley DS, Mohan S (2009). Stem cell antigen-1+ cell-based bone morphogenetic protein-4 gene transfer strategy in mice failed to promote endosteal bone formation.. J Gene Med.

[15] Chada K, Magram J, Raphael K, Radice G, Lacy E (1985). Specific expression of a foreign beta-globin gene in erythroid cells of transgenic mice.. Nature.

[16] Chasis JA, Mohandas N (2008). Erythroblastic islands: niches for erythropoiesis.. Blood.

[17] Pestina TI, Hargrove PW, Jay D, Gray JT, Boyd KM (2009). Correction of murine sickle cell disease using gamma-globin lentiviral vectors to mediate high-level expression of fetal hemoglobin.. Mol Ther.

[18] Cavazzana-Calvo M, Payen E, Negre O, Wang G, Hehir K (2010). Transfusion independence and HMGA2 activation after gene therapy of human beta-thalassaemia.. Nature.

[19] Montini E, Cesana D, Schmidt M, Sanvito F, Ponzoni M (2006). Hematopoietic stem cell gene transfer in a tumor-prone mouse model uncovers low genotoxicity of lentiviral vector integration.. Nat Biotechnol.

[20] Modlich U, Navarro S, Zychlinski D, Maetzig T, Knoess S (2009). Insertional transformation of hematopoietic cells by self-inactivating lentiviral and gammaretroviral vectors.. Mol Ther.

[21] Meng X, Neises A, Su RJ, Payne KJ, Ritter L (2011). Efficient Reprogramming of Human Cord Blood CD34(+) Cells Into Induced Pluripotent Stem Cells With OCT4 and SOX2 Alone.. Mol Ther.

[22] Choi KY, Kim HJ, Lee MH, Kwon TG, Nah HD (2005). Runx2 regulates FGF2-induced Bmp2 expression during cranial bone development.. Dev Dyn.

[23] Mazue G, Bertolero F, Garofano L, Brughera M, Carminati P (1992). Experience with the preclinical assessment of basic fibroblast growth factor (bFGF).. Toxicol Lett.

[24] Nagai H, Tsukuda R, Mayahara H (1995). Effects of basic fibroblast growth factor (bFGF) on bone formation in growing rats.. Bone.

[25] Stier S, Ko Y, Forkert R, Lutz C, Neuhaus T (2005). Osteopontin is a hematopoietic stem cell niche component that negatively regulates stem cell pool size.. J Exp Med.

[26] Nakayama T, Mutsuga N, Tosato G (2007). FGF2 posttranscriptionally down-regulates expression of SDF1 in bone marrow stromal cells through FGFR1 IIIc.. Blood.

[27] Iwaniec UT, Mosekilde L, Mitova-Caneva NG, Thomsen JS, Wronski TJ (2002). Sequential treatment with basic fibroblast growth factor and PTH is more efficacious than treatment with PTH alone for increasing vertebral bone mass and strength in osteopenic ovariectomized rats.. Endocrinology.

[28] Shimada T, Mizutani S, Muto T, Yoneya T, Hino R (2001). Cloning and characterization of FGF23 as a causative factor of tumor-induced osteomalacia.. Proc Natl Acad Sci U S A.

[29] Jonsson KB, Zahradnik R, Larsson T, White KE, Sugimoto T (2003). Fibroblast growth factor 23 in oncogenic osteomalacia and X-linked hypophosphatemia.. N Engl J Med.

[30] de Menezes Filho H, de Castro LC, Damiani D (2006). Hypophosphatemic rickets and osteomalacia.. Arq Bras Endocrinol Metabol.

[31] Juppner H, Wolf M, Salusky IB (2010). FGF-23: More than a regulator of renal phosphate handling?. J Bone Miner Res.

[32] Zhang XB, Schwartz JL, Humphries RK, Kiem HP (2007). Effects of HOXB4 overexpression on ex vivo expansion and immortalization of hematopoietic cells from different species.. Stem Cells.

[33] Martin I, Muraglia A, Campanile G, Cancedda R, Quarto R (1997). Fibroblast growth factor-2 supports ex vivo expansion and maintenance of osteogenic precursors from human bone marrow.. Endocrinology.

[34] Walsh S, Jefferiss C, Stewart K, Jordan GR, Screen J (2000). Expression of the developmental markers STRO-1 and alkaline phosphatase in cultures of human marrow stromal cells: regulation by fibroblast growth factor (FGF)-2 and relationship to the expression of FGF receptors 1–4.. Bone.

[35] Baddoo M, Hill K, Wilkinson R, Gaupp D, Hughes C (2003). Characterization of mesenchymal stem cells isolated from murine bone marrow by negative selection.. J Cell Biochem.

[36] Bianchi G, Banfi A, Mastrogiacomo M, Notaro R, Luzzatto L (2003). Ex vivo enrichment of mesenchymal cell progenitors by fibroblast growth factor 2.. Exp Cell Res.

[37] Nissen LJ, Cao R, Hedlund EM, Wang Z, Zhao X (2007). Angiogenic factors FGF2 and PDGF-BB synergistically promote murine tumor neovascularization and metastasis.. J Clin Invest.

[38] Chaffer CL, Dopheide B, Savagner P, Thompson EW, Williams ED (2007). Aberrant fibroblast growth factor receptor signaling in bladder and other cancers.. Differentiation.

[39] Barde I, Laurenti E, Verp S, Wiznerowicz M, Offner S (2011). Lineage- and stage-restricted lentiviral vectors for the gene therapy of chronic granulomatous disease.. Gene Ther.

[40] Santilli G, Almarza E, Brendel C, Choi U, Beilin C (2011). Biochemical correction of X-CGD by a novel chimeric promoter regulating high levels of transgene expression in myeloid cells.. Mol Ther.

[41] Bergwitz C, Juppner H (2010). Regulation of phosphate homeostasis by PTH, vitamin D, and FGF23.. Annu Rev Med.

[42] Silver J, Naveh-Many T (2010). FGF23 and the parathyroid glands.. Pediatr Nephrol.

[43] Xiao L, Naganawa T, Lorenzo J, Carpenter TO, Coffin JD (2010). Nuclear isoforms of fibroblast growth factor 2 are novel inducers of hypophosphatemia via modulation of FGF23 and KLOTHO.. J Biol Chem.

[44] Martin A, Liu S, David V, Li H, Karydis A (2011). Bone proteins PHEX and DMP1 regulate fibroblastic growth factor Fgf23 expression in osteocytes through a common pathway involving FGF receptor (FGFR) signaling.. FASEB J.

[45] Chen ST, Gysin R, Kapur S, Baylink DJ, Lau KH (2007). Modifications of the fibroblast growth factor-2 gene led to a marked enhancement in secretion and stability of the recombinant fibroblast growth factor-2 protein.. J Cell Biochem.

[46] Yam PY, Li S, Wu J, Hu J, Zaia JA (2002). Design of HIV vectors for efficient gene delivery into human hematopoietic cells.. Mol Ther.

[47] Wang H, Shayakhmetov DM, Leege T, Harkey M, Li Q (2005). A Capsid-Modified Helper-Dependent Adenovirus Vector Containing the β-Globin Locus Control Region Displays a Nonrandom Integration Pattern and Allows Stable, Erythroid-Specific Gene Expression.. Journal of Virology.

[48] Beyer I, Li Z, Persson J, Liu Y, van Rensburg R (2011). Controlled extracellular matrix degradation in breast cancer tumors improves therapy by trastuzumab.. Mol Ther.

[49] Sheng MH, Baylink DJ, Beamer WG, Donahue LR, Lau KH (2002). Regulation of bone volume is different in the metaphyses of the femur and vertebra of C3H/HeJ and C57BL/6J mice.. Bone.

