# Supplementary figures and images for: Erythroid Promoter Confines FGF2 Expression to the Marrow after Hematopoietic Stem Cell Gene Therapy and Leads to Enhanced Endosteal Bone Formation
Source: PLoS One. 2012 May 18;7(5):e37569. doi: 10.1371/journal.pone.0037569 (PMC3356341; doi:10.1371/journal.pone.0037569)

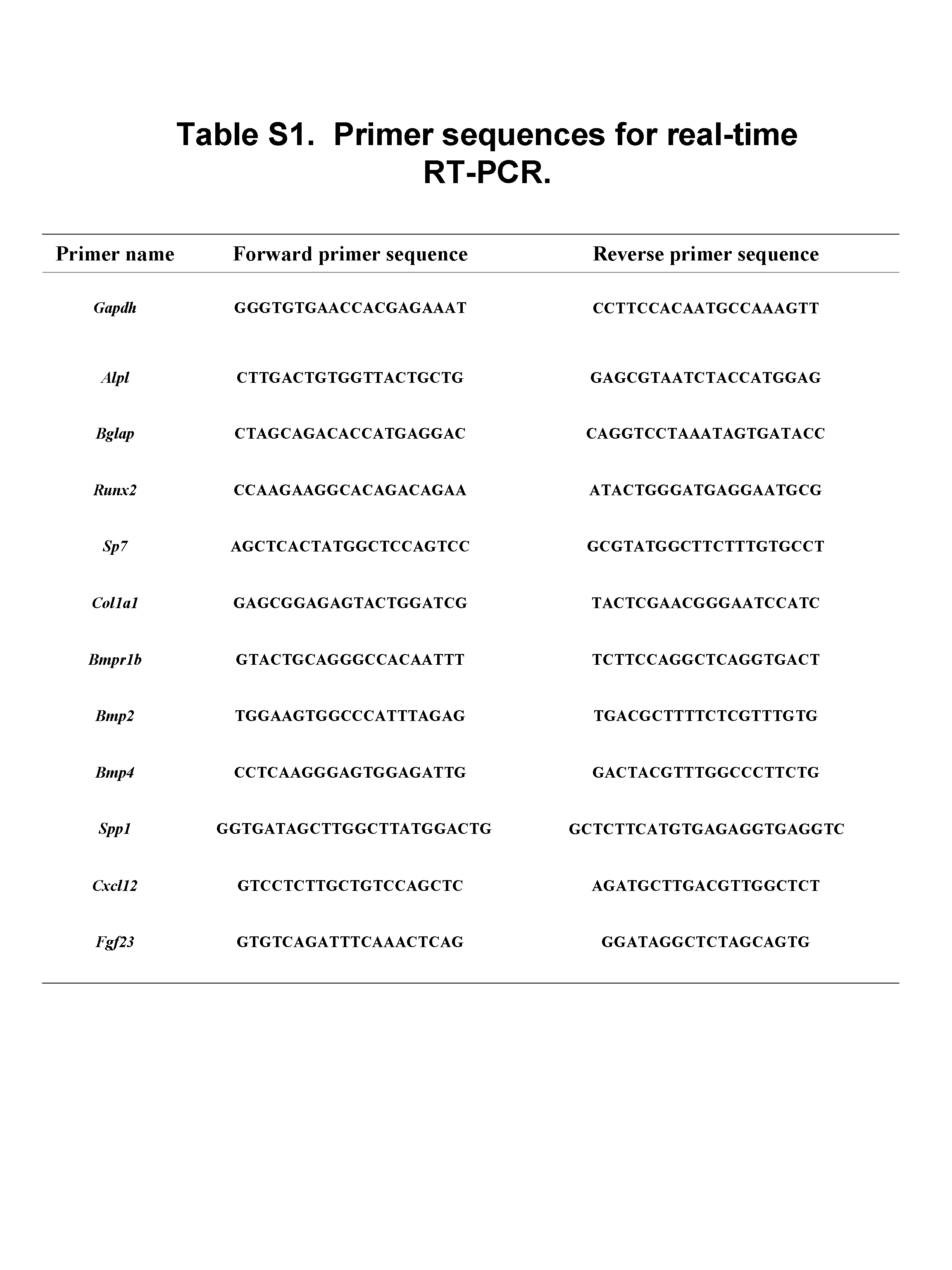

Supplement: Table S1 — Primer sequences for real-time RT-PCR. (TIF) [file pone.0037569.s001.tif]
